# Upward movement of IS4 and IIIS4 is a rate-limiting stage in Ca_v_1.2 activation

**DOI:** 10.1007/s00424-016-1895-5

**Published:** 2016-10-29

**Authors:** Stanislav Beyl, Annette Hohaus, Stanislav Andranovits, Eugen Timin, Steffen Hering

**Affiliations:** Department of Pharmacology and Toxicology, University of Vienna, Althanstrasse 14, 1090 Vienna, Austria

**Keywords:** Calcium channel, Voltage sensor, Pore, Mutational analysis, Gating, Electrophysiology, Patch clamp, Heart

## Abstract

In order to specify the role of individual S4 segments in Ca_V_1.2 gating, charged residues of segments IS4-IVS4 were replaced by glutamine and the corresponding effects on activation/deactivation of calcium channel currents were analysed. Almost all replacements of charges in IS4 and IIIS4 decreased the slope of the Boltzmann curve of channel activation (activation curve) while charge neutralisations in IIS4 and IVS4 did not significantly affect the slope. S4 mutations caused either left or rightward shifts of the activation curve, and in wild-type channels, these S4 mutations hardly affected current kinetics.

In slowly gating pore (S6) mutants (G432W, A780T, G1193T or A1503G), neutralisations in S4 segments significantly accelerated current kinetics. Likewise in wild type, charge replacements in IS4 and IIIS4 of pore mutants reduced the slope of the activation curves while substitutions of charges in IIS4 and IVS4 had less or no impact. We propose a gating model where the structurally different S4 segments leave their resting positions not simultaneously. Upward movement of segments IS4 and (to a lesser extend) IIIS4 appear to be a rate-limiting stage for releasing the pore gates. These segments carry most of the effective charge for channel activation. Our study suggests that S4 segments of Ca_V_1.2 control the closed state in domain specific manner while stabilizing the open state in a non-specific manner.

**Electronic supplementary material:**

The online version of this article (doi:10.1007/s00424-016-1895-5) contains supplementary material, which is available to authorized users.

## Introduction

Voltage-gated calcium channels (Ca_V_) mediate calcium influx into muscle, endocrine and neuronal cells, thereby regulating key cellular functions such as contraction, excitability, sensory processes, release of hormones and neurotransmitters and gene expression [7, 8, 28]. Recently reported structural data on the Ca_V_1.1 complex revealed a high degree of structural homology of the pore-forming α1-subunit to other voltage-gated ion channels [33]. The α1-subunits of Ca_V_ are composed of four homologous concatenated domains, each formed by six transmembrane segments (S1–S6). The voltage-sensing domains (segments S1–S4) communicate with the channel pore (formed by S5, S6 and the connecting pore loop) [6, 33]. Every third residue of the S4 segments carries a charged amino acid (arginine or lysine). It is believed that pore opening of Ca_V_ is initiated by the voltage-dependent upward movement of positively charged S4 while its downward movement during repolarisation forces the channel to close. Structural studies suggest that the voltage sensors and pore gates are located at some distance from each other and may function as essentially independent units. This is indirectly supported by the fact that some K^+^ channels have a pore domain but no voltage-sensing domain while voltage-sensing domains without a pore domain regulate phosphatase activity and proton permeation [15, 21, 25].

The ‘low voltage activated’ Ca_V_3 and ‘high voltage activated’ Ca_V_1/ Ca_V_2 carry comparable numbers of charges on their S4 segments while activating at significantly different voltages. For comparison, the *shaker* Kv channel has seven charged residues in each S4 (a total of 28 charged residues, see [9]); the bacterial NavAb [27] carries four S4 charges and a natural concatameric sodium channel such as Nav1.4 has a total number of gating charges ranging between four in IS4 and eight in IVS4 (GenBank: AIE46146.1). The functional or evolutionary background to such differences is currently not understood.

Structural changes (point mutations) in Ca^2+^ channels may influence activation and inactivation properties (e.g. [10, 17, 22, 32]). We have recently shown that Ca_V_1.2 comprising a IIS4 segment where all arginines and lysine were replaced by glutamine (resulting in IIS4_N_) open and close with kinetics very similar to the wild type [1]. A similar observation was made for a Ca_V_1.2 channel in which four out of five IS4 charges were neutralised [2]. At first glance, these data suggested that IS4 and IIS4 have no significant impact on Ca_V_1.2 activation.

**Fig. 1:**
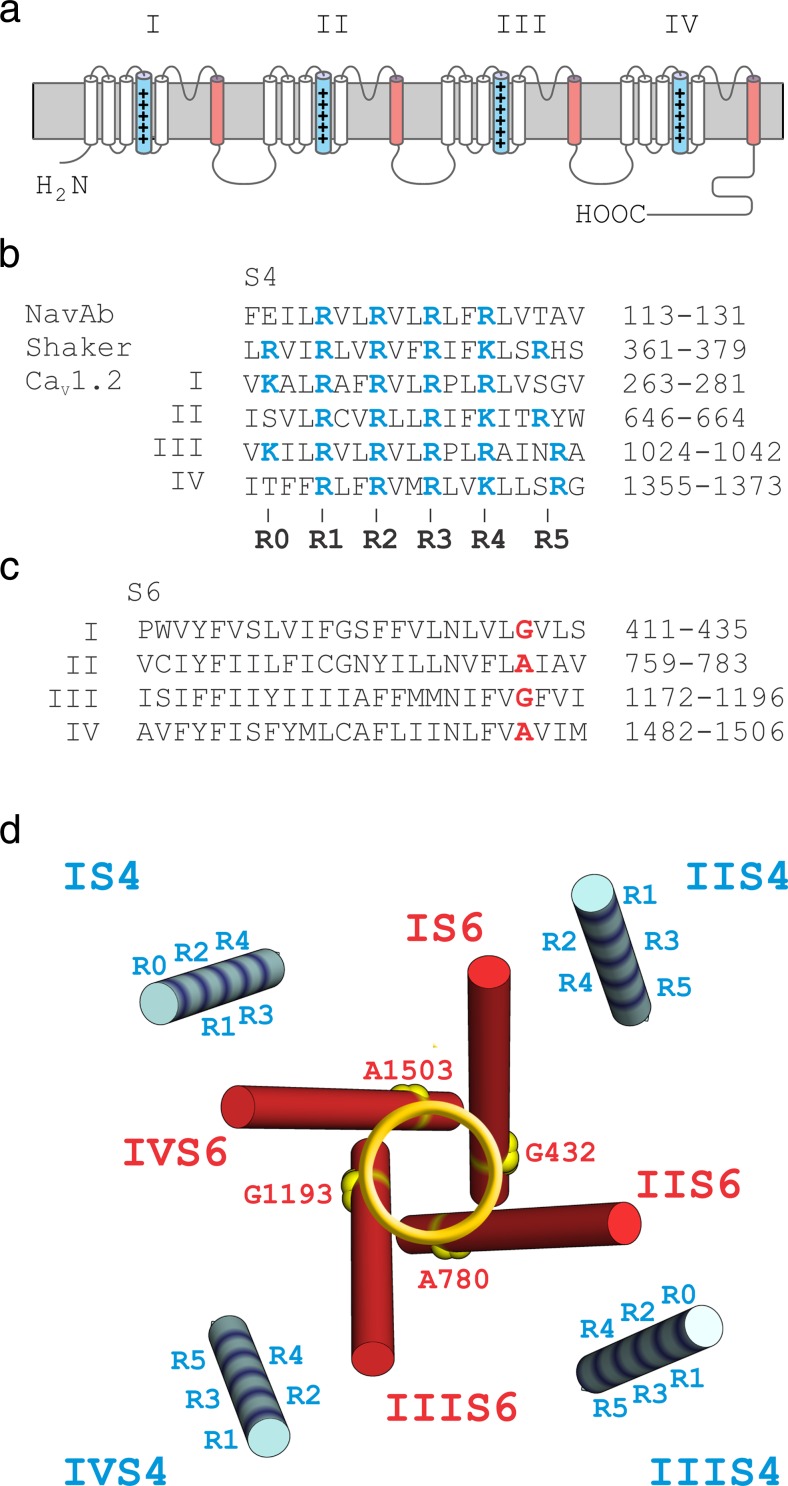
Voltage-sensing S4 and pore-forming S6 segments of Ca_V_1.2. **a** Schematic representation of pore-forming α_1_ subunit of Ca_V_1.2 with S4 (*blue*) and S6 (*red*) segments highlighted. **b**, **c** Alignments of S4 (**b**) and S6 (**c**) segments of Ca_V_1.2 (accession number, P15381) compared with other channel types. Charged residues are shown in *blue*. A ring of glycine or alanine residues on pore forming IS6–IVS6 (‘GAGA’ G432, A780, G1134, A1503) is highlighted in *red*. **d** Voltage-sensing S4 (*blue*) and pore-forming S6 (*red*) segments of Ca_V_1.1 (pdb: 3JBR) channel shown as structural representation of corresponding segments of Ca_V_1.2 α1 subunit. GAGA residues are assumed to participate in stabilisation of the closed gates [1]. Charged S4 residues and the 'GAGA ring' in the pore are *highlighted*. (Colour figure online)

However, an important role of IIS4 in Ca_V_1.2 gating became evident if channel kinetics were slowed by specific point mutations on the S6 gates (a ring of alanines and glycines: G432W (IS6), A780T (IIS6), G1193T (IIIS6), A1503G (IVS6), Fig. 1c, designated as ‘GAGA mutations’ [1]). When charged IIS4 residues in these mutants were replaced by glutamines, activation/deactivation was accelerated and the activation curves of these constructs were shifted to the right [1].

Remarkably, IIS4_N_ affected pore mutations in all four S6 gates. We thus proposed a ‘cooperative gating model’ where the voltage-dependent movement of a S4 segment pushing a single S6 into its closing position increases the probability of all four S6 segments to occupy the closed state [1].

Evidence that individual subunits gate cooperatively, rather than independently, comes also from previous studies on potassium channels [4, 30, 31, 36]. In concatameric channels such as Ca_V_1.2, we face a more complex situation with four structurally different domains comprising structurally different S4 segments carrying different numbers of charged residues.

An important role of the voltage sensors of domains I and III but not II and IV in gating of Ca_V_1.2 was previously reported by Yamaguchi et al. [34]. Evidence for individual roles of S4 segments in Ca_V_1.2 gating comes also from the work of Pantazis et al. [26] who tracked the movement of the individual voltage sensors using voltage-clamp fluorometry. Their data illustrate that kinetics of fluorescence quenching of labelled IIS4 and IIIS4 are comparable with the activation and deactivation of the ion current, suggesting that these segments are predominantly involved in Ca_V_1.2 activation, with IS4 and IVS4 making smaller contributions [26]. Here, we investigate the individual roles of S4 segments in gating of Ca_V_1.2 gating by neutralising a ‘maximal possible’ number of charges in all four S4 segments.

## Materials and methods

### Mutagenesis

Substitutions in S4 and S6 segments of the Ca_V_1.2 α_1_-subunit (GenBank™ X15539) were introduced using the QuikChange® Lightning Site-Directed Mutagenesis Kit (Stratagene) with mutagenic primers according to the manufacturer’s instructions. All constructs were checked by restriction site mapping and sequencing.

### Cell culture and transient transfection

Human embryonic kidney tsA-201 cells were grown at 5 % CO_2_ and 37 °C to 80 % confluence in Dulbecco’s modified Eagle’s/F-12 medium supplemented with 10 % (*v*/*v*) foetal calf serum and 100 units/ml penicillin/streptomycin. Cells were split with trypsin/EDTA and plated on 35-mm Petri dishes (Falcon) at 30–50 % confluence ∼16 h before transfection. Subsequently, tsA-201 cells were co-transfected with complementary DNAs (cDNAs) encoding wild-type or mutant Ca_V_1.2 α_1_ subunits with auxiliary β_2a_ [29] as well as α_2_-δ_1_ [11] subunits and GFP to identify transfected cells.

The transfection of tsA-201 cells was performed using the FuGENE HD Transfection Reagent (Roche) following standard protocols. tsA-201 cells were used until passage number 15. No variation in channel gating related to different cell passage numbers was observed.

### Ionic current recordings and data acquisition

Barium currents (I_Ba_) through voltage-gated Ca^2+^ channels were recorded at 22–25 °C by patch clamping [13] using an Axopatch 200A patch clamp amplifier (Axon Instruments, Foster City) 36–48 h after transfection. The extracellular bath solution (in mM: BaCl_2_ 5, MgCl_2_ 1, HEPES 10, choline-Cl 140) was titrated to pH 7.4 with methanesulfonic acid. Patch pipettes with resistances of 1 to 4 MΩ were made from borosilicate glass (Clark Electromedical Instruments, UK) and filled with pipette solution (in mM: CsCl 145, MgCl_2_ 3, HEPES 10, EGTA 10), titrated to pH 7.25 with CsOH. All data were digitised using a Digidata 1200 interface (Axon Instruments, Foster City), smoothed by means of a four-pole Bessel filter and saved to disc. Current traces (100-ms) were sampled at 10 kHz and filtered at 5 kHz; tail currents were sampled at 50 kHz and filtered at 10 kHz. Leak currents were subtracted either digitally using average values of scaled leakage currents elicited by a 10-mV hyperpolarising pulse or electronically by means of an Axopatch 200 amplifier (Axon Instruments, Foster City). Series resistance and offset voltage were routinely compensated for. The pClamp software package (Version 10.0 Axon Instruments, Inc.) was used for data acquisition and preliminary analysis. Microcal Origin 7.0 was used for analysis and curve fitting.

The voltage dependence of activation was determined from I–V curves and fitted to

$$ {m}_{\infty }=\frac{1}{1+ \exp \frac{V_{0.5,\mathrm{act}}-V}{k_{\mathrm{act}}}} $$

The time courses of current activation and deactivation were fitted to a mono-exponential function

$$ I(t)=A\cdot \exp \left(\frac{t}{\tau}\right)+C $$

where *I*(*t*) is the current at time *t*; *A* is the amplitude coefficient; *τ* is the time constant and *C* the steady-state current. Data are given as mean ± S.E. Time constants were plotted versus voltage (e.g. Fig. 4d). The left branch of the bell-shaped curve of the time constants corresponds to channel deactivation and the right branch to the activation (see [16]). At voltages where channel activation and deactivation overlap (peak of the bell-shaped dependence), the data are given as averaged values of both time constants.

To minimise effects of inactivation on the estimation of the rate of channel activation and deactivation, the α_1_ subunit of Ca_V_1.2 was co-expressed with the auxiliary β_2a_ subunit known to substantially slow the inactivation kinetics [14]. Furthermore, in order to avoid Ca^2+^-dependent inactivation, the experiments were performed with Ba^2+^ as charge carrier.

## Results

We have previously reported that complete neutralisation of positive charges in each segment IS4-IVS4 in the α_1_ subunit of Ca_V_1.2 resulted in only one functional channel construct (IIS4_N_, [1]). No inward currents were recorded if the charged residues in IS4, IIIS4 and IVS4 (IS4_N_, IIIS4_N_ and IVS4_N_) were substituted by glutamines (see also [1]).

**Fig. 2:**
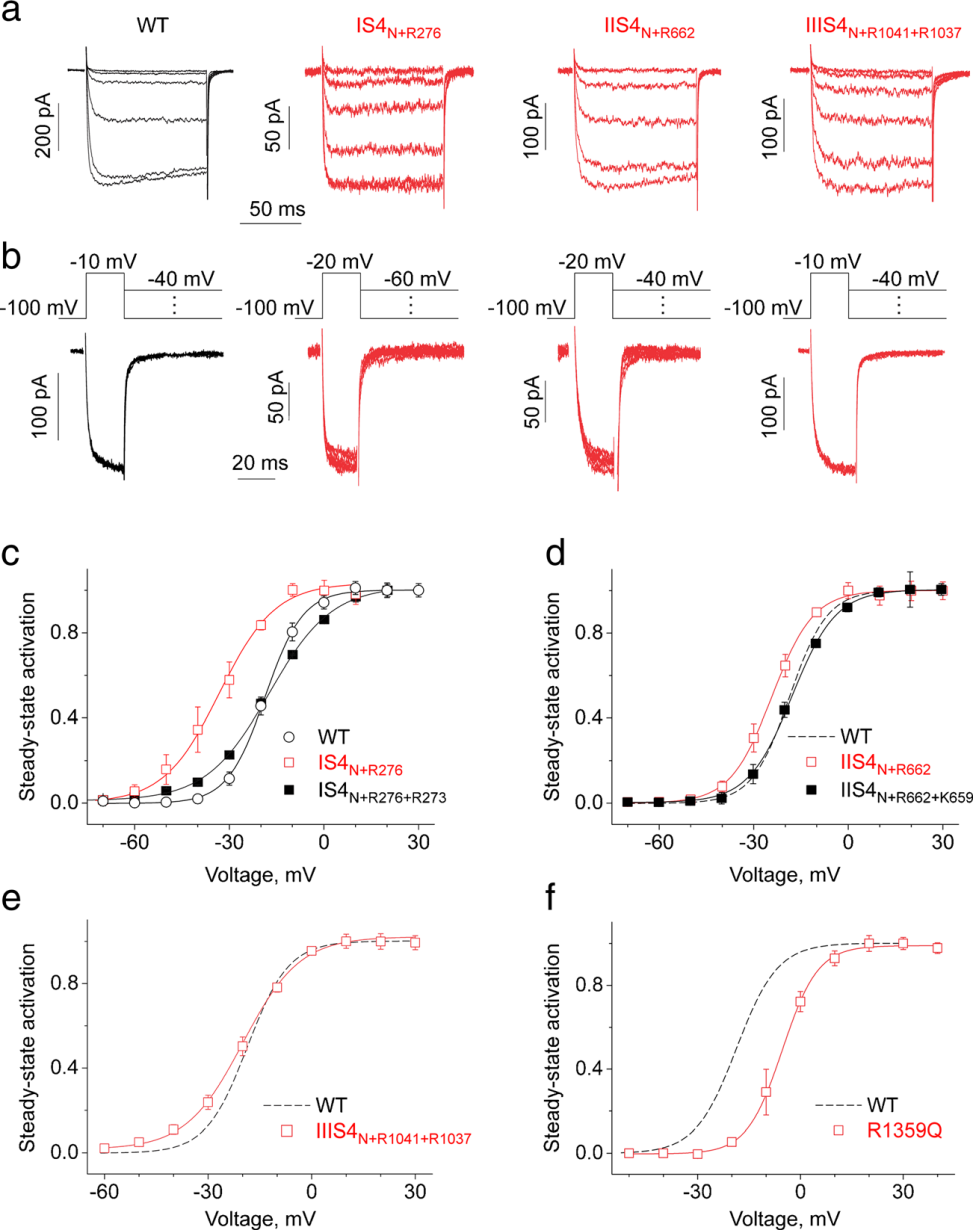
Modulation of Ca_V_1.2 gating by S4 charge neutralisations. Activation (**a**) and deactivation (**b**, tail current) of *I*_Ba_ through wild-type and mutant channel constructs with partially neutralised S4 segments (IS4_N+R276_, IIS_4N+R662_, IIIS4_N+R1041+R1037_). The deactivation was measured by applying the voltage steps to different potentials after a short (20 ms) activating pulse (**b**). Note that neutralisation of S4 charges has minor effects on activation and deactivation kinetics of Ca_V_1.2. **c**–**f** Averaged activation curves of wild-type, IS4_N+R276_, IS4_N+R276+R273_, IIS_4N+R662_, IIS_4N+R662+R652_, IIIS4_N+R1041+R1037_ and R1359Q channel constructs. The slope of IS4_N+R267_ (**c**) and IIIS4_N+R1041+R1037_ (**e**) activation curves was significantly reduced (*k*_act_ = 5.9 ± 0.9 mV in WT vs 8.5 ± 0.6 mV and 9.9 ± 0.7 mV in IS4_N+R267_ and IIIS4_N+R1041+R1037_ respectively)

Constructs IS4_N+R276_ and IIS4_N+R662_ carrying a single S4 charge in the lowest positions (R5, Figs. 1b and 2b) formed functional channels (Fig. 2); no currents were recorded after transfection with IIIS4_N+R1041_ and IVS4_N+R1372_, Table 1). Two charged residues in IIIS4 (R5, R4, IIIS4_N+R1041+R1037_) and four charged residues (IVS4 constructs R1359Q or R1365Q) in IVS4 were essential for formation of functional channels (Figs. 1 and 2). In the second part of this study, we use slowly-gating pore mutants as tools to analyse the domain-specific impact of S4 segments on Ca_V_1.2 current kinetics.

**Table 1:** Midpoints and slope factors (*k*) of the activation curves and estimated effective charge (αZ)

Mutant	V_0.5_, mV	*k*_act_, mV	αZ
WT	−18.6 ± 0.8 (*n* = 7)	5.9 ± 0.9	4.27 ± 0.65
G432W^b^	−33.7 ± 0.6 (8)	4.7 ± 0.4	5.36 ± 0.46
A780T^b^	−44.0 ± 0.6 (5)	5.1 ± 0.6	4.94 ± 0.58
G1193T^b^	−49.8 ± 1.0 (7)	5.1 ± 0.6	4.94 ± 0.58
A1503G^b^	−41.4 ± 0.8 (5)	5.8 ± 0.5	4.34 ± 0.37
IS4 neutralisations			
IS4_N_	No detectable current		
K264Q/R267Q/R270Q/R273Q (IS4_N+R276_)	−33.1 ± 1.1 (5)	8.5 ± 0.6	2.96 ± 0.21
K264Q/R267Q/R270Q (IS4_N+R276+R273_)	−16.3 ± 0.7 (4)	9.9 ± 0.7	2.54 ± 0.18
G432W/R267Q	−11.9 ± 1.0 (4)	8.5 ± 0.8	2.96 ± 0.28
G432W/IS4_N+R276_	No detectable current		
A780T/R267Q	−28.5 ± 1.3 (4)	9.6 ± 0.7	2.62 ± 0.19
A780T/IS4_N+R276_	−42.7 ± 1.1 (4)	11.9 ± 1.0	2.12 ± 0.18
A780T/K264Q/R267Q/R270Q (A780T/IS4_N+R276+R273_)	−33.5 ± 0.6 (4)	11.5 ± 0.6	2.19 ± 0.11
G1193T/R267Q	−28.6 ± 0.9 (5)	9.1 ± 0.5	2.77 ± 0.15
G1193T/IS4_N+R276_	No detectable current		
A1503G/R267Q	−28.5 ± 0.6 (5)	9.1 ± 0.4	2.77 ± 0.12
A1503G/IS4_N+R276_	−41.1 ± 0.6 (5)	8.7 ± 0.5	2.89 ± 0.17
IIS4 neutralisations			
IIS4_N_^a^	−17.5 ± 0.8 (11)	6.7 ± 0.7	3.76 ± 0.39
R650Q/R653Q/R656Q/K659Q (IIS4_N+R662_)	−23.9 ± 1.0 (6)	6.3 ± 0.7	4.00 ± 0.44
R650Q/R653Q/R656Q (IIS4_N+R662+K659_)	−18.2 ± 0.7 (5)	7.2 ± 0.6	3.50 ± 0.29
R650Q/R653Q	-25.1 ± 0.9 (5)	5.7 ± 0.6	4.42 ± 0.47
G432W/R656Q	−17.9 ± 0.9 (5)	6.0 ± 0.7	4.20 ± 0.49
G432W/IIS4_N+R662_	−31.0 ± 0.6 (5)	8.4 ± 0.8	3.00 ± 0.29
G432W/IIS4_N_^a^	−19.3 ± 0.7 (8)	5.8 ± 0.7	4.34 ± 0.52
A780T/R650Q	−30.7 ± 0.3 (4)	5.7 ± 0.3	4.42 ± 0.23
A780T/R653Q	−30.2 ± 0.6 (5)	5.3 ± 0.3	4.75 ± 0.27
A780T/R656Q	−33.5 ± 0.5 (5)	6.5 ± 0.3	3.88 ± 0.18
A780T/K659Q	−32.0 ± 1.3 (4)	7.1 ± 0.8	3.55 ± 0.40
A780T/R662Q	−34.0 ± 0.7 (5)	5.4 ± 0.3	4.67 ± 0.26
A780T/IIS4_N+R662_	−33.6 ± 0.6 (4)	6.3 ± 0.3	4.00 ± 0.19
A780T/IIS4_N+R650_	−39.4 ± 0.8 (4)	6.1 ± 0.6	4.13 ± 0.41
A780T/IIS4_N_^a^	−32.0 ± 0.7 (10)	5.1 ± 0.7	4.94 ± 0.68
G1193T/R650Q	−39.0 ± 0.9 (5)	6.8 ± 0.6	3.71 ± 0.33
G1193T/R653Q	−34.0 ± 0.6 (6)	6.6 ± 0.5	3.82 ± 0.29
G1193T/R656Q	−38.3 ± 0.8 (5)	5.0 ± 0.5	5.04 ± 0.50
G1193T/K659Q	−40.3 ± 0.9 (4)	4.9 ± 0.5	5.14 ± 0.52
G1193T/R662Q	−40.2 ± 0.4 (6)	5.7 ± 0.3	4.42 ± 0.23
G1193T/IIS4_N+R662_	−38.0 ± 0.8 (5)	6.1 ± 0.6	4.13 ± 0.41
G1193T/IIS4_N_^a^	−20.8 ± 0.7 (5)	7.5 ± 0.6	3.36 ± 0.27
A1503G/R656Q	−26.6 ± 0.7 (5)	7.3 ± 0.5	3.45 ± 0.24
A1503G/IIS4_N+R662_	−25.3 ± 1.0 (6)	7.7 ± 0.6	3.27 ± 0.25
A1503G/IIS4_N_^a^	−19.8 ± 0.7 (6)	7.5 ± 0.7	3.36 ± 0.31
IIIS4 neutralisations			
IIIS4_N_	No detectable current		
K1025Q/R1028Q/R1031Q/R1034Q/R1037 (IIIS4_N+R1041_)	No detectable current		
K1025Q/R1028Q/R1031Q/R1034Q (IIIS4_N+R1041+R1037_)	−19.7 ± 0.9 (5)	8.0 ± 0.5	3.15 ± 0.20
R1034Q	−23.4 ± 0.6 (5)	6.5 ± 0.4	3.82 ± 0.23
A780T/K1025Q/R1028Q/R1031Q/R1034Q	−43.6 ± 0.6	9.3 ± 0.4	2.69 ± 0.12
A780T/K1025Q/R1028Q	−34.7 ± 0.5 (7)	7.7 ± 0.3	3.29 ± 0.11
A780T/R1034Q	−33.5 ± 0.6 (4)	6.1 ± 0.5	4.14 ± 0.31
G1193T/K1025Q/R1028Q/R1031Q/R1034Q	−41.8 ± 0.6	8.2 ± 0.6	3.09 ± 0.21
A1503G/R1034Q	−27.4 ± 0.5 (4)	7.1 ± 0.3	3.56 ± 0.13
IVS4 neutralisations			
IVS4_N_	No detectable current		
R1359Q/R1362Q/R1365Q/K1368Q (IVS4_N+R1372_)	No detectable current		
R1359Q/R1362Q/R1365Q	No detectable current		
R1359Q/R1362Q	No detectable current		
R1359Q	−6.0 ± 1.0 (5)	5.0 ± 0.6	5.04 ± 0.60
R1365Q	−5.8 ± 1.0 (5)	5.6 ± 1.0	4.47 ± 0.79
G1193T/K1025Q/R1028Q	−35.8 ± 0.7 (7)	8.7 ± 0.6	2.89 ± 0.20

Numbers of experiments are indicated in parentheses

^a^Data from [1]

^b^Data from [10]

### Neutralisation of IS4–IIIS4 charges has little effects on kinetics of Ca_V_1.2 activation

**Fig. 3:**
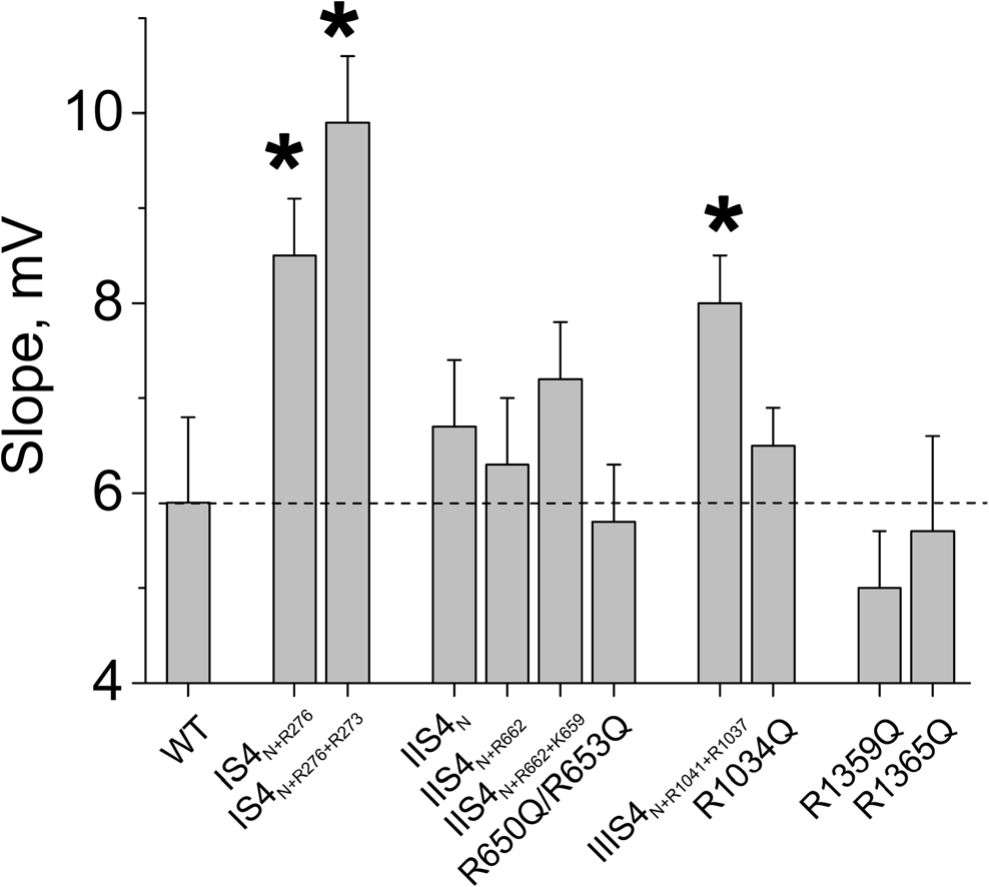
Neutralisations in IS4–IVS4 differently affect the slope of the Ca_V_1.2 activation curve. Compared to wild-type Ca_V_1.2, slope factors of the activation curves were significantly (*p < 0.05) increased by charge neutralisations in IS4 and IIIS4 but not by neutralisations of charged residues in segments IIS4 and IVS4

Figure 2 illustrates the activation/deactivation properties of channel construct IS4_N+R276_. Replacing four out of five charged IS4 residues by glutamine did not affect current kinetics (see current kinetics of IS4_N+R276_ in Fig. 2a, b), but caused a significant shift of the activation curve to negative voltages (from −18.6 ± 0.8 mV in WT to −33.1 ± 1.1 mV) and a significant reduction of the slope of the Boltzmann curve (from k_act_ = 5.9 ± 0.9 to 8.5 ± 0.6 mV, see Table 1, Fig. 3). Adding a second charge resulting in IS4_N+R276+R273_ also decreased the slope of the Boltzmann distribution and shifted the curve to the right (*k* = 9.9 ± 0.7, Fig. 2c, see Table 1).

A similar study was performed with segment IIS4. However, adding either one or two charges to IIS4_N_ (constructs IIS4_N+R662_, IIS4_N+R662+K659_, Fig. 1b) induced only minor shifts of the activation curves and affected neither current kinetics nor the slope of the Boltzmann distribution (Figs. 2 and 3, see also Table 1).

Transfection with cDNA of construct IIIS4_N+R1041_ did not result in functional channels. Ca_V_1.2 carrying the lower two IIIS4 charges (R5 and R4: R1041 and R1037) was functional. The slope of the Boltzmann curve of IIIS4_N+R1041+R1037_ was significantly reduced compared to wild-type channels (Figs. 2 and 3, Table 1). Currents activated and deactivated with kinetics similar to the wild type (Fig. 2a, b).

### Gating effects of single charge neutralisation in segment IVS4

Neutralisation of either all five (R1–R5), the upper four (R1–R4), the upper three (R1–R3) or even two (R1 and R2, Fig. 1b) charges of IVS4 did not result in conducting channel constructs (Table 1). Thus, four charged residues (e.g. constructs R1359Q or R1365Q) had to be present in IVS4 to form functional channels. R1365Q (corresponding to neutralisation of R3, Fig. 1) shifted the Boltzmann distribution towards more depolarised voltages (V_0.5_ = −5.8 ± 1.0 mV) without affecting the slope of the curve (Table 1, Fig. 3). A similar observation was made for construct R1359Q (Table 1, Fig. 2f).

### Slowly gating pore mutants confirm different impacts of S4 segments on Ca_V_1.2 gating

In general, charge neutralisation in IS4–IVS4 did not affect current kinetics (Fig. 2a, b) which prevented conclusions about S4-mediated stabilisations of either open and/or closed channel conformations.

In order to further elucidate the individual effects of segments IS4 and IIIS4 on gating, we made use of four pore mutations on S6 gates (G432W (IS6), A780T (IIS6), G1193T (IIIS6), A1503G (IVS6)). In these mutants, the maximum of the bell-shaped dependence of the time constants on voltage was substantially increased while extreme values (at large depolarisation and hyperpolarisation) remained unchanged [1, 10]. We anticipated that the use of these S6 mutants would enable kinetic studies of domain-specific S4 effects.

**Fig. 4:**
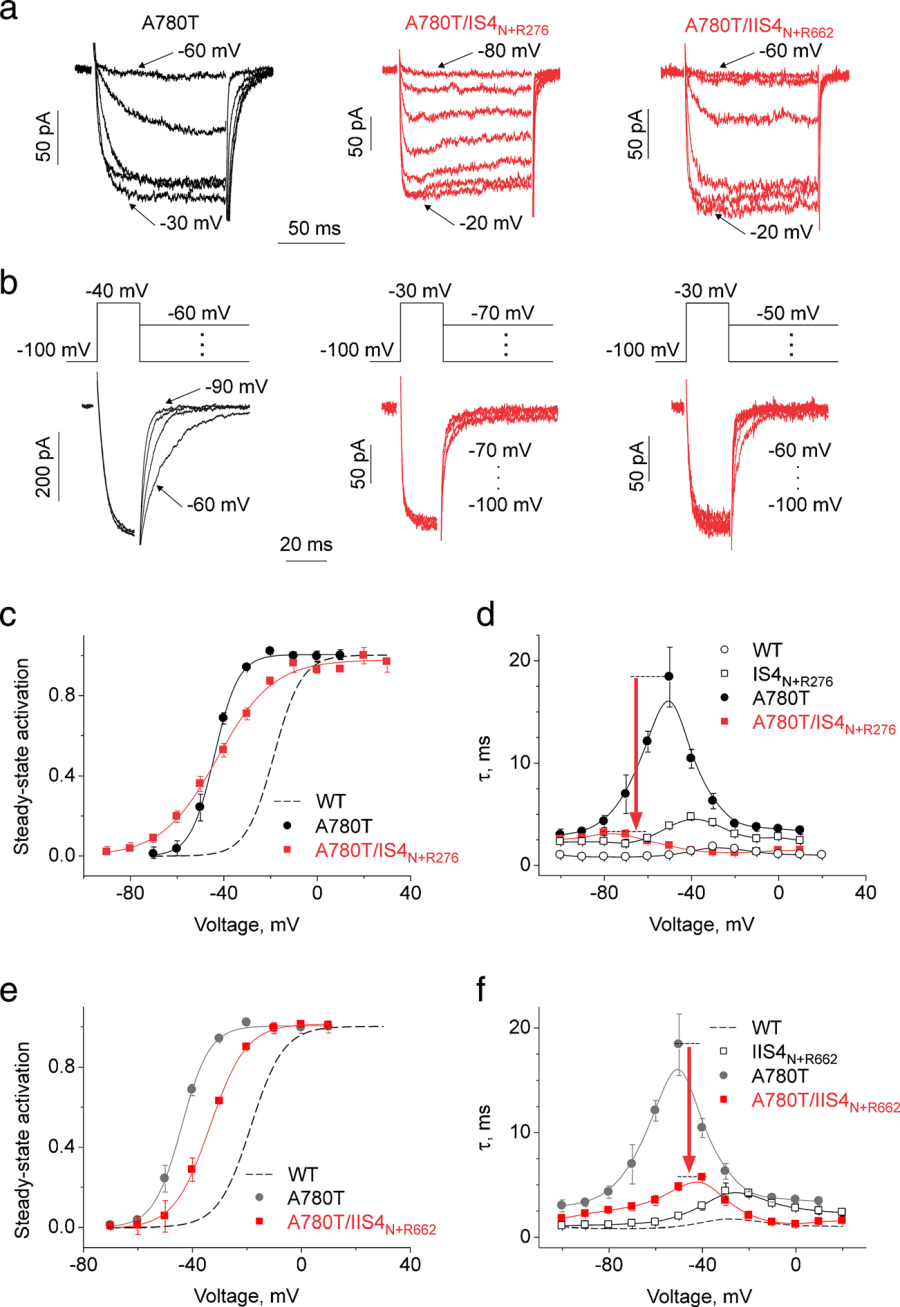
Modulation of pore mutant A780T by IS4 and IIS4 carrying a single charge (IS4_N+R276_ and IIS4_N+R662_). Activation (**a**) and deactivation (**b**, tail current) of *I*_Ba_ through pore mutant A780T and corresponding constructs with partially neutralised segments IS4 (IS4_N+R276_, four out of five IS4 charges are substituted by glutamines) and IIS4 (IIS4_N+R267_, four out of five IIS4 charges are substituted by glutamines). The deactivation was measured by applying the voltage steps to different potentials after a short (20 ms) activating pulse (**b, voltage protocol above current traces**). Partial neutralisation of IS4 or IIS4 charges had minor effects on activation and deactivating kinetics (compare with corresponding traces on Fig. 2) but caused a significant acceleration of current kinetics of the slowly gating A780T (constructs A780T/IS4_N+R267_ and A780T/IIS4_N+R662_, *arrows* on **d** and **f**. **c**, **e** Averaged activation curves of WT, A780T, A780T/IS4_N+R267_ and A780T/IIS4_N+R662_ channels. The slope of A780T/IS4_N+R267_ was significantly reduced (from *k*_act_ = 5.1 ± 0.6 mV in A780T to *k*_act_ = 11.9 ± 1.0 mV in A780T/IS4_N+R267_). The slope of A780T/IIS4_N+R662_ activation curve was not changed while the activation curve was shifted by +10.3 ± 0.8 mV (see Table 1). Analogous gating disturbances induced by mutations in the conserved ring of small residues (either glycines or alanines, Fig. 1d) and the effect of S4 charge neutralisation are presented on Figs. S1, S2, S3, and S4 (Supplemental Materials) and also Table 1. **d**, **f** Voltage-dependent time constants of channel activation/deactivation. *Red arrows* highlight the﻿ ﻿acceleration﻿﻿ of current kinetics when﻿ A780T﻿ was combined with a ‘neutralised’ S4 segment. (Colour figure online)

Indeed, as shown in Fig. 4a, kinetics of the slowly gating construct A780T were substantially accelerated by IS4_N+R276_ (construct A780T/IS4_N+R276_, see also Table 1 and Fig. S2 in Supplemental Materials for other constructs). Thus, removing four out of five charges from IS4 reduced the time constant of activation/deactivation at the peak of the bell-shaped curve from 18.4 ± 2.9 ms in A780T to 3.1 ± 0.2 ms in A780T/IS4_N+R276_ (Fig. 4d). Acceleration of tail currents by introduction of IS4_N+R276_ into A780T underlines an important role of IS4 charges in stabilisation of the open channel pore.

Furthermore, combining IS4_N+R276_ with A780T significantly decreased the slope of the activation curve (slope factor increased from 5.1 ± 0.6 to 11.9 ± 1.0 mV, *p* < 0.05, Table 1, compare to Fig. 2) without significantly shifting the midpoint of the curves (see also Fig. S2 in Supplemental Materials for construct A1503T/IS4_N+R276_ and Table 1).

Contributions of IIIS4 charges to Ca_V_1.2 activation (see also Fig. 2c) are illustrated by construct A780T/IIIS4_N+R1041+R1037_. Neutralisation of four out of six IIIS4 charges in pore mutant A780T substantially reduced the slope of the activation curve (Fig. 6). Additionally, neutralisation of IIIS4 charges on pore mutant A780T substantially accelerated deactivation (from *τ*_max_ = 18.4 ± 2.9 in A780T to 5.2 ± 0.5 ms in A780T/IIIS4_N+R1041+R1037_), highlighting a role of this segment in stabilisation of the open channel state.

A contrasting picture was observed for A780T/IIS4_N+R662_ (Fig. 4). IIS4 carrying only a single arginine at the lowest position (R662) shifted the activation curve of A780T by +10.3 ± 0.8 mV to the right without, however, affecting the slope of the curve (Fig. 4e). Figure 4f illustrates the acceleration of current kinetics of A780T caused by IIS4 neutralisation (IIS4_N+R662_). A similar observation was made for combinations of IIS4_N+R662_ with three other pore mutations (G432W (IS6), G1193T (IIIS6), A1503G (IIIS6); see Figs. S2, S3, and S4 in Supplemental Materials, Table 1). The observed shifts of the activation curve ranged between +2.7 ± 0.8 mV (G432W/IIS4_N+R662_) and +16.1 ± 1.3 mV (A1503G/IIS4_N+R662_). No significant effect of a partially charged IIS4 on the slope of the activation curve was observed for most of these constructs (Table 1). In some pore mutants, charge neutralisations in segment IIS4 reduced, however, the activation slope (e.g. G432W/IIS4_N+R662_ and A780T/K659Q) suggesting peculiarities of the pore–S4 interplay in these constructs. Effects of charge neutralisations in IIS4 on gating of pore mutants A780T and G1193T are summarised in Fig. S1 (Supplemental Material). Interestingly, removal of the outermost arginine in IVS4 segment of the Ca_V_3.1 channel affects amplitude but not voltage dependence of gating current [20].

### Neutralisation of single charges in segments IS4 and IIS4 differently affect gating

**Fig. 5:**
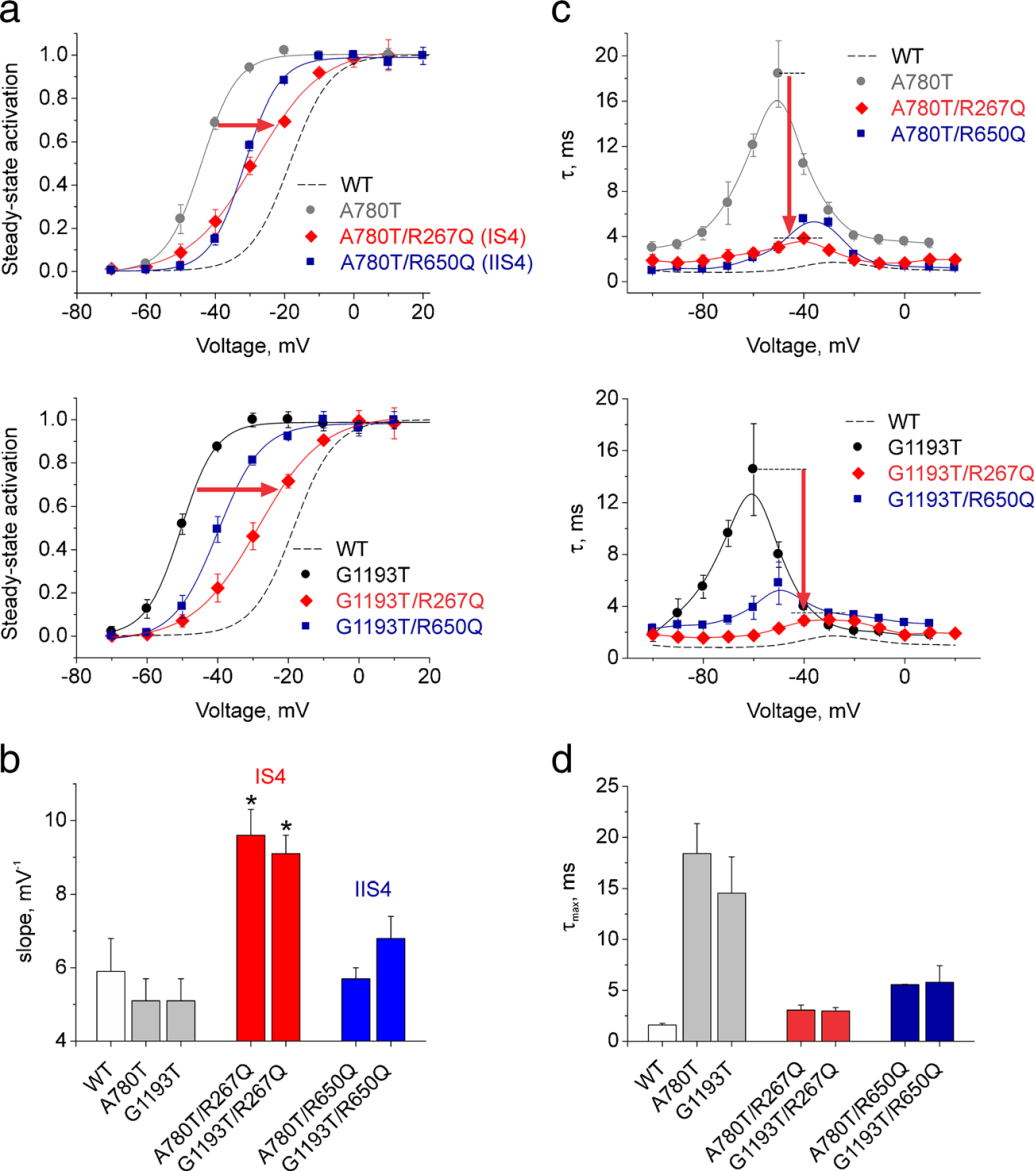
Neutralisation of single IS4 and IIS4 charges differently affects gating of pore mutants A780T and G1193T. **a** Averaged activation curves of WT; A780T and G1193T and constructs A780T/R267Q, G1193T/R267Q (IS4), A780T/R650Q, G1193T/R650Q (IIS4). *Red arrows* illustrate the rightward shift of the activation curves upon neutralisation of IS4 R267 (R267Q) and IIIS4 R267 (R267Q) charges. Note that neutralisation of single IS4 and IIIS4 charges caused significant reductions in slope of the activation curves (*red curves*, see also Table 1). **b** Slope factors of the activation curves of pore mutants (*grey bars*) are significantly increased by single charge neutralisation in IS4 (*red bars*) but not in IIS4 (*blue bars*). See also Table 1 and Figs. S2, S3, and S4 (Supplemental Materials) for other constructs. **c**, **d** Voltage-dependent time constants of channel activation/deactivation (**c**) and the slowest activation time constant at the peak of the bell-shaped curve (*τ*_max_) (**d**). Note that voltage-sensor neutralisations in IS4 (*red*) or IIS4 (*blue*) significantly accelerate the kinetics of ‘slowly’ gating pore mutants. (Colour figure online)

Figure 5 illustrates that neutralisation of even a single charge in segments IS4 and IIS4 substantially affects gating. In IS4, we replaced arginine in positions 267 (corresponding to R1) and in IIS4 in position 650 (R1, Fig. 1) by glutamine. Both S4 mutations shifted the activation curves of pore mutants A780T and G1193T towards the wild type. Activation of A780T/R267Q was shifted by +16 mV and A780T/R650Q by +13 mV. The activation curves of G1193T/R267Q and G1193T/R650Q were shifted by +20 and +11 mV respectively (Fig. 5a, b).

Neutralisation of a single arginine in IS4 did, however, result in a significant (*p* < 0.05) reduction of the slope which is in line with observations made for other IS4 mutations (Figs. 2 and 4, Table 1). Substitution of IS4 or IIS4 charges substantially accelerated channel kinetics in pore mutants A780T and G1193T (Fig. 5c, d).

### S4 segments carry different numbers of effective charges

**Fig. 6:**
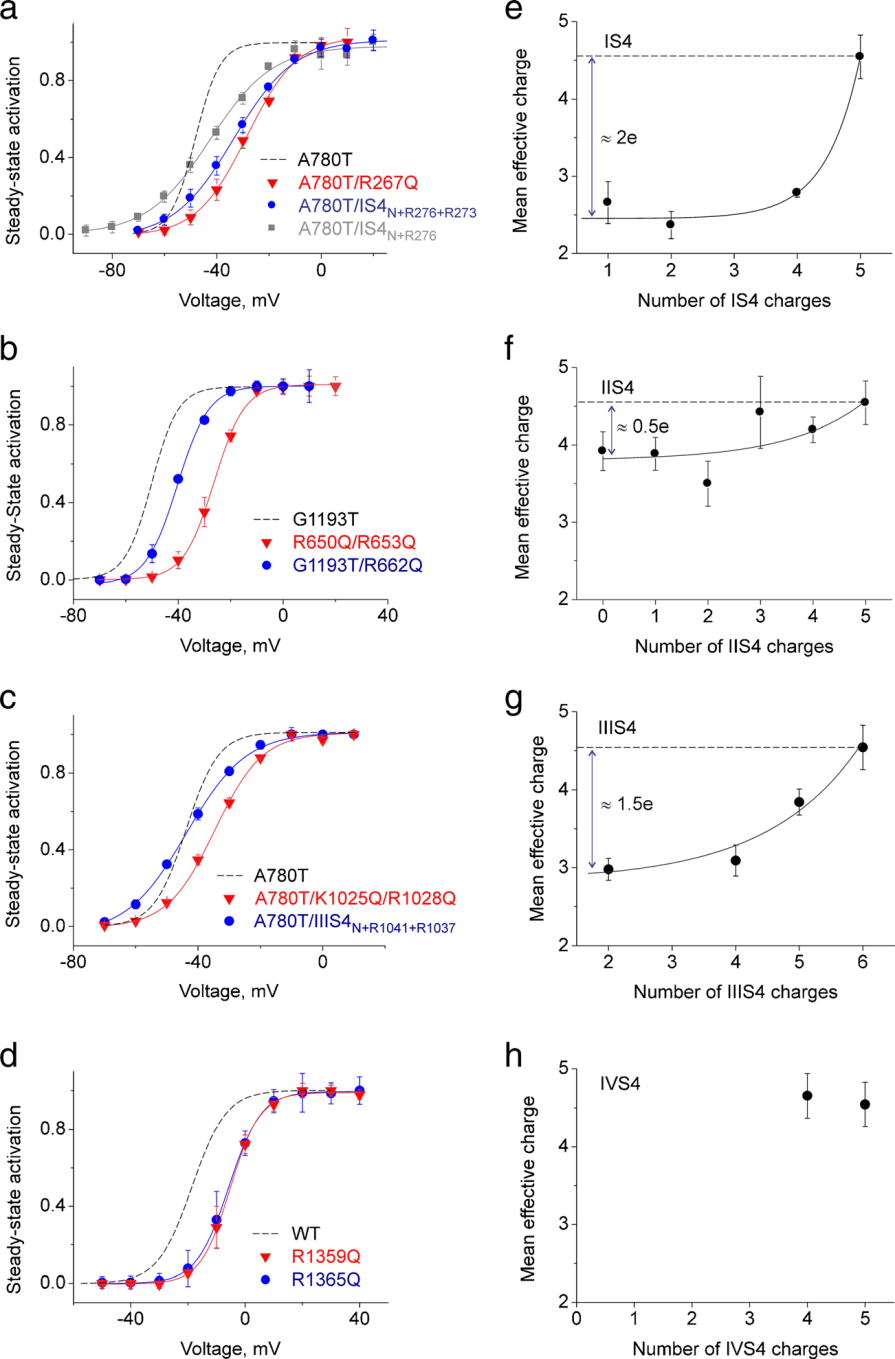
Modulation of gating by neutralisation of S4 segments. **a**–**d** Averaged activation curves of wild-type and indicated mutant channels. Activation curves of constructs with reduced IS4 or IIIS4 charges are shifted to more negative potentials, and the slope factors of the activation curve of channels with partially charged IS4 or IIIS4 are reduced (see also Table 1). **e**–**h** Mean effective charges of all constructs (wild-type Ca_V_1.2 and pore mutants) were estimated from the slopes of the activation curves (Table 1) and plotted versus the number of charged residues in IS4 (**e**), IIS4 (**f**), IIIS4 (**g**) and IVS4 (**h**). To enable ‘back extrapolation’, data (**e**–**h**) were fitted to a non-linear regression. Back extrapolation of the exponential function yields an apparent mean effective charge of about two elementary units for IS4 (**e**), about 0.5 elementary units for IIS4 (**f**) and ≈1.5 elementary units for IIIS4 (**g**). Choosing an exponential function for analysis does not imply that S4 charges make an exponential contribution to the activation slope

Introducing more than one charge into IS4_N_ (e.g. A780T/IS4_N+R276+R273_) shifted the activation curve towards more depolarised voltages and reduced the slope (Table 1, Fig. 6), an effect that was also evident for constructs IS4_N+R276_ and IS4_N+R276+R273_ (compare with Fig. 2). However, charge neutralisation in wild-type Ca_V_1.2 induced smaller shifts of the Boltzmann curve than in ‘GAGA’ pore mutants (Table 1). Thus, segment IS4 containing four out of five charges (R267Q) shifted the curve even further to the right (V_0.5_ (A780/IS4_N+R276_) −42.7 ± 1.1 < V_0.5_ (A780/IS4_N+R276+R273_) −33.5 ± 0.6 < V_0.5_ (A780/ R267Q) −28.5 ± 1.3 mV, *p* < 0.05) and significantly accelerated gating (Table 1). The slope of activation curves ranged from *k*_act_ = 9.6 ± 0.7 mV in A780T/R267Q to 11.9 ± 1.0 mV in A780T/IS4_N+R276_ compared to *k*_act_ = 5.1 ± 0.6 mV in A780T (see also Table 1). In Fig. 6, we have plotted the effective charges calculated from the activation curves

$$ \alpha Z=\frac{RT}{F}\cdot \frac{1}{k_{act}} $$

against the total number of IS4 charges. Fitting of the data points to a nonlinear regression and back extrapolation of this curve to the Y-intercept yield αZ = 2.5 ± 0.1, resulting in the reduction of effective charges by approximately two elementary charges. A similar analysis for IIIS4 neutralisation yield αZ = (Fig. 6g).

Different reductions of effective charge caused by complete S4 neutralisation (Fig. 6; Δ(αZ)_IS4_ = (αZ)_control_ − (αZ)_IS4N_ = 2.1 ± 0.3, Δ(αZ)_IIS4_ = 0.6 ± 0.4, Δ(αZ)_IIIS4_ = 1.7 ± 0.4) confirm different roles of S4 segments in channel opening and closure: segments IS4 and IIIS4 directly affect channel opening while IIS4 affected the stability of open and closed states.

If more than one arginine in IVS4 was replaced by glutamine, the resulting constructs were not functional (Table 1). Neutralisation of only one charge (R1359Q or R1365Q) did not, however, reduce the effective charge, which clearly distinguishes IVS4 from IS4 and IIIS4 (see Fig. 6). Moreover, the sum of effective charge reduction caused by neutralisation of the first three S4 segments Δ(αZ)_IS4_ + Δ(αZ)_IIS4_ + Δ(αZ)_IIIS4_ = 4.4 ± 0.7 was statistically not different from the control (αZ)_control_ = 4.5 ± 0.3. Taken together, these data suggest that segment IVS4 plays only a minor, if any, role in channel opening, which is in line with the work of Pantazis et al. [26].

## Discussion

A calcium channel construct with completely neutralised segment IIS4 (IIS4_N_) activates and deactivates with kinetics similar to wild-type Ca_V_1.2. In specific pore mutations (GAGA mutations), IIS4_N_, however, substantially accelerates current kinetics [1]. According to Zagotta et al. [36], channel activation involves (i) charge movement in four domains followed by (ii) a ‘concerted’ step of pore opening. Thus, alterations of channel opening kinetics could result from either (i) or (ii). Multiple investigations support the view that in this chain of events, the concerted pore opening is a rate-limiting stage. Thus, current kinetics of wild-type channels and construct IIS4_N_ are likely to be determined by the concerted step of pore opening (ii) and not by the charge movement (i) [18, 23, 31, 36].

In slowly gating pore mutants, the decelerated pore opening is even ‘more rate limiting’. However, in these constructs, IIS4_N_ has been shown to modify rate constants of pore opening and closure (see [2]). Current kinetics of pore mutations in all four S6 segments were significantly accelerated by IIS4_N_ which led us to conclude that IIS4 stabilises the open conformation by modulating a cooperative pore gating mechanism [1].

Here, we investigate the role of segments IS4–IVS4 in gating of Ca_V_1.2 in more detail by studying the effects of gradual charge insertion into neutralised S4 (S4_N_) of different domains on activation/deactivation kinetics and the Boltzmann distribution (steady-state activation curve).

### IS4 and IIIS4 contribute most of the effective charge to the gate unlocking step of channel activation

Functional channels were formed with four out of five IS4 charges (IS4_N+R276_), completely neutralised IIS4 (IIS4_N_, [1]), two out of six IIIS4 charges (IIIS4_N+R1041+R1037_) and four out of five IVS4 charges (IVS4 constructs R1359Q or R1365Q). Effects of charge neutralisation in all four S4 segments on current kinetics were negligible (Fig. 2). However, significant effects on the slope of the activation curve observed for constructs IS4_N+R276_ and IIIS4_N+R1041+R1037_ suggest that these segments play a particular role in channel opening.

Individual impacts of IS4, IIS4 and IIIS4 on Ca_V_1.2 gating become even more evident in the studied pore mutants (Fig. 1c). These slowly gating Ca_V_1.2 constructs are apparently an excellent experimental platform for analysing the effects of even single S4 charges on Ca_V_1.2 current kinetics (e.g. Figs. 4 and 5). Figures 2 and 4 illustrate that IS4_N+R276_ (IS4 carries only one arginine, R5) and also A780T/IS4_N+R276_ combining the pore mutation A780T with IS4_N+R276_ both display a reduced slope of the activation curve. Adding more than one charge to IS4_N_ gradually increased the slope of the activation curve (Figs. 2 and 5a), suggesting a more efficient closure/activation of the channel gate. Slope reductions caused by neutralisation of IS4 and IIIS4 charges were observed for all S6 mutants (Fig. 5, Table 1).

In order to quantify the impact of different S4 segments, we systematically analysed the activation curves of Ca_V_1.2 constructs containing different numbers of charged residues in segments IS4–IVS4 (Table 1).

Previously, we have shown that the position of the curve on the voltage axis predominantly reflects a ratio of the rate constants of pore opening and closure [3, 35]. The slope of the Boltzmann curve is determined by the product of the charge (Z) required to lock/unlock the pore and the fraction of the membrane potential (α) that this charge has to cross. Normally, αZ is significantly smaller than the total charge of S4 segments, suggesting that the distance the voltage sensor has to cross to trigger opening of the pore gates is short. The apparent charge movement measured from a Boltzmann curve is always much less than the number of charges that move across the membrane (approximately 4–8 vs 12–16 [24]).

In Ca_V_1.2, the dependence of the effective charge on the actual number of charged residues in IS4 can be fitted by an exponential function (Fig. 6e). Backward extrapolation to the Y-intercept yields an apparent effective charge of about two elementary units for this segment. In other words, the neutralisation of IS4 charges is equivalent to a decrease of the effective channel charge by two units. This led us to conclude that IS4 contributes 2 out of 4.5 effective charges to channel opening. A similar analysis of IIIS4 mutants revealed a smaller contribution of charges of this segment to activation (≈1.5 elementary units, Fig. 6).

Instead, five charges of IIS4 contribute only about 0.5–1 elementary units (Fig. 6f). Hence, neutralisation of any number of IIS4 charges has only a small effect on the effective charge (Table 1, Fig. 6), suggesting a minor role of this segment in the channel opening step. Our kinetic study suggests that IIS4 contributes efficiently to stabilisation of the open channel state illustrated by acceleration of current deactivation upon charge neutralisation (Fig. 4). This is in line with Pantazis et al. and our previous finding that stabilisation of the open state (decrease of the rate constant of closure) leads to pronounced deceleration of deactivation without inducing changes in the slope of the steady-state activation curve [1].

Remarkably, removal of single IVS4 charges does not change the effective charge of channel gating (no reduction in slope was observed, Fig. 6). It was, however, not possible to reduce the number of S4 charges below four, which limits this analysis.

### S4 segments modulate a cooperative pore gating mechanism

Complete neutralisation of segment IIS4 affects gating perturbations in all four pore-forming S6 segments [1]. Hence, neutralisation of IIS4 charges (IIS4_N_) shifts the activation curve of S6 mutants in all four domains I–IV (i.e. mutations in positions G432/A780/G1193/A1503, Fig. 1). In other words, the voltage-sensing S4 segment in domain II affected gating in domains I, II, III and IV. This finding was interpreted as an indication for cooperative assembling of all four gating structures in the closed state at rest. We further hypothesised that the closed conformation is additionally locked (stabilised) by downward movement of voltage-sensing segments [1].

Here, we show that this is not a unique property of IIS4 and that other S4 segments also affect gating disturbances caused by mutations on different S6 segments. Construct (IS4_N+R276_) in combination with A780T (domain II) or A1503G (domain IV) accelerated kinetics of these constructs (A780T/IS4_N+R276_, Fig. 2, Fig. S2 in Supplemental Materials, as previously shown for IIS4 [1]). Similar observations were made for IIIS4 charge neutralisations in combination with pore mutations in IIS6 (A780T), IIIS6 (G1193T) and IVS4 (A1503G) (Table 1). Taken together, these data support a gating model where segments IS4, IIS4 and IIIS4 in Ca_V_1.2 modulate the stability of cooperatively opening and closing channel gates.

### Gating effects of partially and completely charged IIS4

Interestingly, IIS4 carrying four out of five charges (R656Q) induced similar acceleration of current kinetics and similar shifts of the activation curves as IIS4 carrying only a single charged residue (IIS4_N+R662_, Fig. 4 and Fig. S1 in Supplemental Materials). A ‘fully charged’ IIS4 was more efficient in stabilising the open channel pore than a partially charged IIS4. We speculate that a partially charged IIS4 fails to reach its optimal ‘key in the lock’ position for stabilisation of the open state while IIS4 carrying all five charges does.

### Design principles of the Ca_V_1.2 gating machinery

If we translate the Hodgkin-Huxley model [16] for potassium currents into current terminology, all four gating units (S4 segments) must be in an activated position before the channel can open.

This hypothesis was supported by data from Horn et al. [18] (see [19] for review) showing that immobilisation of only one S4 segment in its down position prevents *shaker* activation. Furthermore, potassium channel closure (deactivation) seems to require only one S4 in its resting position [12].

Our data suggest a similar gating mechanism for Ca_V_1.2. Changes in the slope of the activation curve (Boltzmann distribution) were predominantly induced by charge neutralisations in segments IS4 and IIIS4 (Figs. 2 and 3). Both segments carry most of the effective charge for channel activation (Fig. 5). If S4 segments in Ca_V_1.2 move not simultaneously, then the upward movement of IS4 and (to a lesser extend) IIIS4 would be rate limiting for the pore opening step. This assumption is in line with Pantazias et al. [26], showing that S4 segments of different Ca_V_1.2 domains move with different speed during activation.

**Fig. 7:**
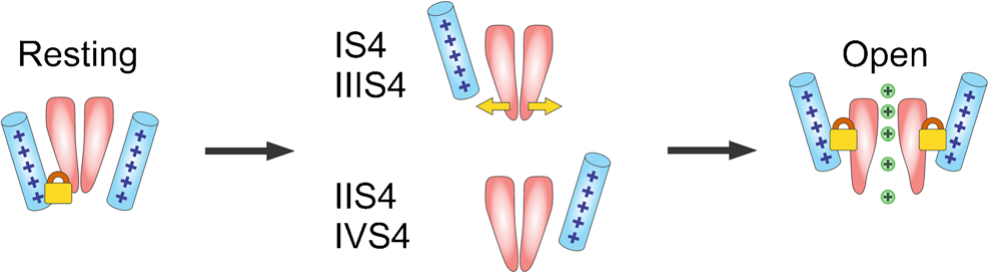
Different impacts of S4 segments on Ca_V_1.2 activation. *Cartoon* illustrating the hypothetical role of IS4 and IIIS4 in locking the channel pore in the closed conformation. Their upward movement correspondingly ‘releases’ the activation gates (enables a concerted pore opening) and stabilises the pore in the open conformation. Both S4 segments together contribute ≈90 % to the effective charge (Fig. 6e, g). Segment IIS4 contributes only 10 % of the effective charge for activating the channel (Fig. 6f). All four S4 stabilise the open state. IS4 and IIIS4 determine the voltage dependence to both the opening (‘unlocking the closed state’) and the closing of gate structures.

Our studies with the slowly gating pore mutants suggest that IS4–IIIS4 all contribute to stabilisation of the open channel conformation (Fig. 7). This is evident from the acceleration of the channel deactivation caused by charge neutralisation of the different S4 segments (Figs. 4 and 5; see also Figs. S1 and S2 in Supplemental Material).

Specific impacts of S4 segments on channel gating have previously been reported for voltage-gated sodium channels where immobilisation of a single S4 (IIS4) prevents channels from opening while photocross-linking of IVS4 affects both activation and inactivation [18]. Capes et al. [5] reported that in Nav1.4, activation of IVS4 alone is sufficient for fast inactivation to occur. In Ca_V_1.2, the role of different S4 segments in voltage-dependent inactivation has yet to be determined.

Another principal finding is that conformational changes caused by even single charge neutralisations in IS4, IIS4 and IIIS4 (Figs. 5) are not restricted to modulation of a single counterpart gate structure but are extended to gating structures in other domains. These data suggest that the stability of the open channel state is particularly sensitive to the number of charges in different S4 segments. Cooperative interactions between gates in the closed and open conformations (see also Supplemental Materials) are essential elements of the Ca_V_1.2 gating machinery. Our results strongly suggest that, in addition to electrostatic interactions between charged residues and the membrane electric field, interactions between S4 and cooperative gating of pore-forming S6 segments determine the voltage dependence of Ca_V_1.2 activation.

## Electronic supplementary material

ESM 1 (DOC 827 kb)
