# Supplementary material for: Upward movement of IS4 and IIIS4 is a rate-limiting stage in Cav1.2 activation
Source: Pflugers Arch. 2016 Oct 29;468(11):1895–907. doi: 10.1007/s00424-016-1895-5 (PMC5138263; doi:10.1007/s00424-016-1895-5)
Supplement: Supplementary file 1 — (DOC 827 kb) [file 424_2016_1895_MOESM1_ESM.doc]

**Upward movement of IS4 and IIIS4 is a rate limiting stage in Ca_v_1.2 activation**

**Stanislav Beyl, Annette Hohaus, Stanislav Andranovits, Eugen Timin, Steffen Hering***

From the Department of Pharmacology and Toxicology, University of Vienna,

Althanstrasse 14, 1090 Vienna, Austria

##### Supplementary Material

***Little positional specific effects of IIS4 charge neutralisations***

Ca_V_1.2 constructs carrying either one (IIS4_N+R662_) or four IIS4 charges (R650Q, Fig. S1) displayed similar gating characteristics. This was characteristic for IIS4 while an increasing number of charged residues in IS4 induced gradual shifts of the activation curves and increase of the slopes (compare Fig. 5A and S1).

In order to analyse whether similar effects of A780T/IIS4_N+R662_ and A780T/R650Q result from a positional specific impact of R650 we subsequently replaced single IIS4 charges at different positions by glutamine (Fig. S1). Neutralisation of R650, R653, R656, K659 or R662 in IIS4 shifted the activation curves of G1193T (ΔV~+12mV) and the other GAGA mutants between +10.0±0.9 mV and +15.8±1.1 mV in the depolarizing directions (Figs. S1-S2). In general, these different IIS4 constructs induced similar accelerations of current kinetics and similar shifts of the activation curve. Bar graphs in Fig. S1 illustrate changes in the voltage of half maximal activation of G1193T/IIS4_N_ and G1193T (and A780T/IIS4_mut_ and A780T) where only a single IIS4 charge in different positions was neutralised and a single charge (R662) was introduced into IIS4_N_ (G1193T/IIS4_N+R662_). Remarkably, IIS4 carrying a single charge or, alternatively, four out of five charges induced similar shifts of the curve. Fig. S1 D compares the activation/deactivation time constants of different G1193/IIS4_mut_ and A780T/ IIS4_mut_ constructs. Again, a similar acceleration was observed for all constructs. The shifts of activation curves and changes in current kinetics induced by S4 charge neutralizations in other GAGA mutants are summarised in Figs. S2-S4.

***Supplemental Figures***

FIGURE S1


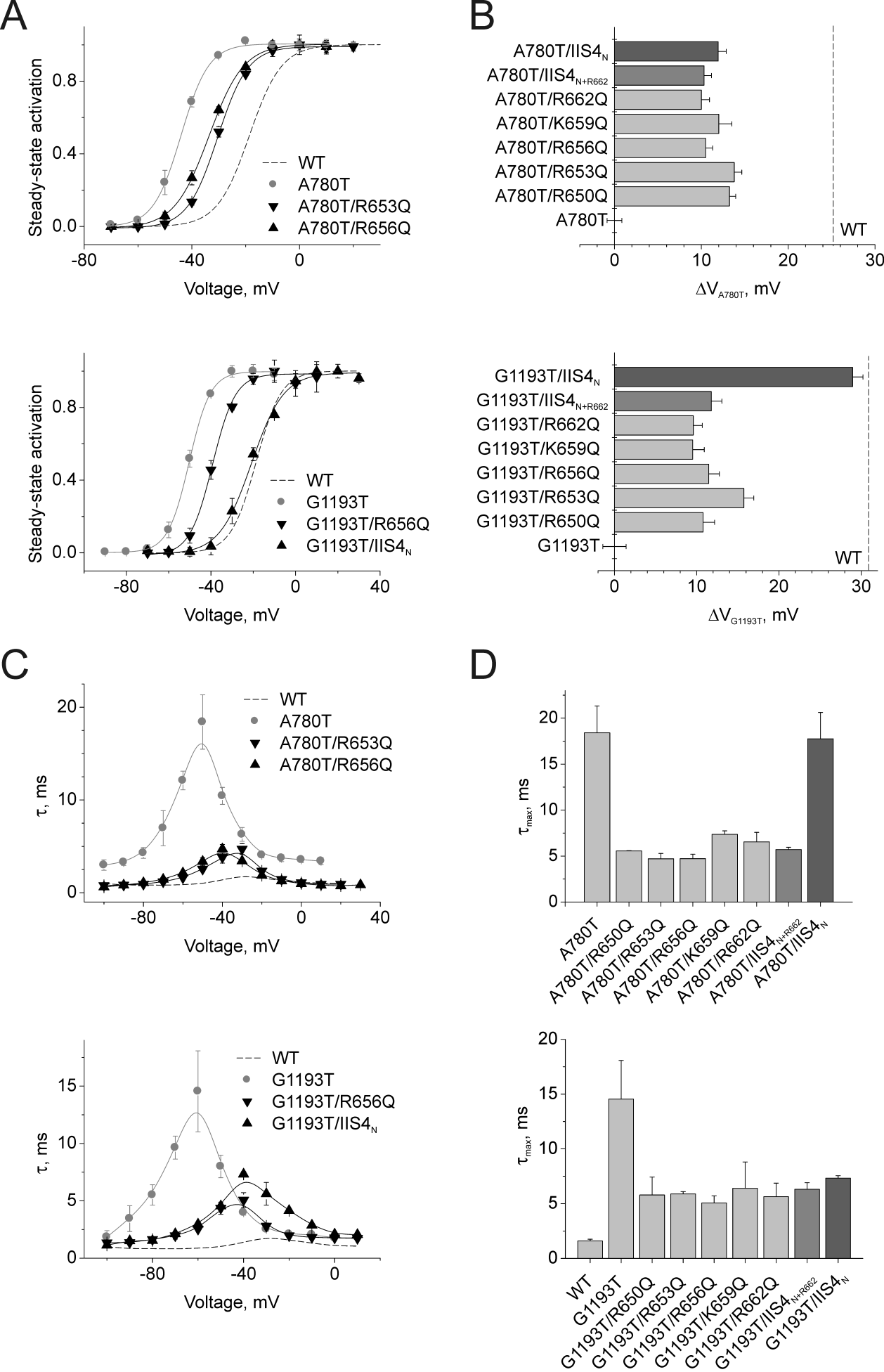


**Fig. S1 Charge neutralisation in IIS4 rescues gating disturbances caused by A780T or G1193T**

A and B, Effect of charge neutralisation on steady-state activation of A780T (segment IIS6) or G1193T (segment IIIS6). The shifts of activation curves are given in relation to A780T or G1193T. Dashed lines represent the position of WT steady-state curve relative to GAGA mutations. See also Table 1 and Figures S2-S4 (supplemental material) for results with other GAGA mutants.

C and D, voltage-dependent time constants of channel activation/deactivation (C) and τ_max_ (D) of studied constructs. IIS4 neutralisations significantly accelerate the “slowly” gating GAGA mutants.

**IS4 and IIS4 contribute to open state stability**

Studies with the slowly gating pore mutants (e.g. A780T, G1193T, see Figs. S1, S2) reveiled that IS4 and IIS4 contribute to stabilization of the open channel conformation. This is evident from the acceleration of the channel deactivation caused by charge neutralization of IS4 or IIS4 segments (see Fig. S1 C,D; Fig. S2 C,D; Fig. S3 C,D; Fig. S4 C,D).

FIGURE S2


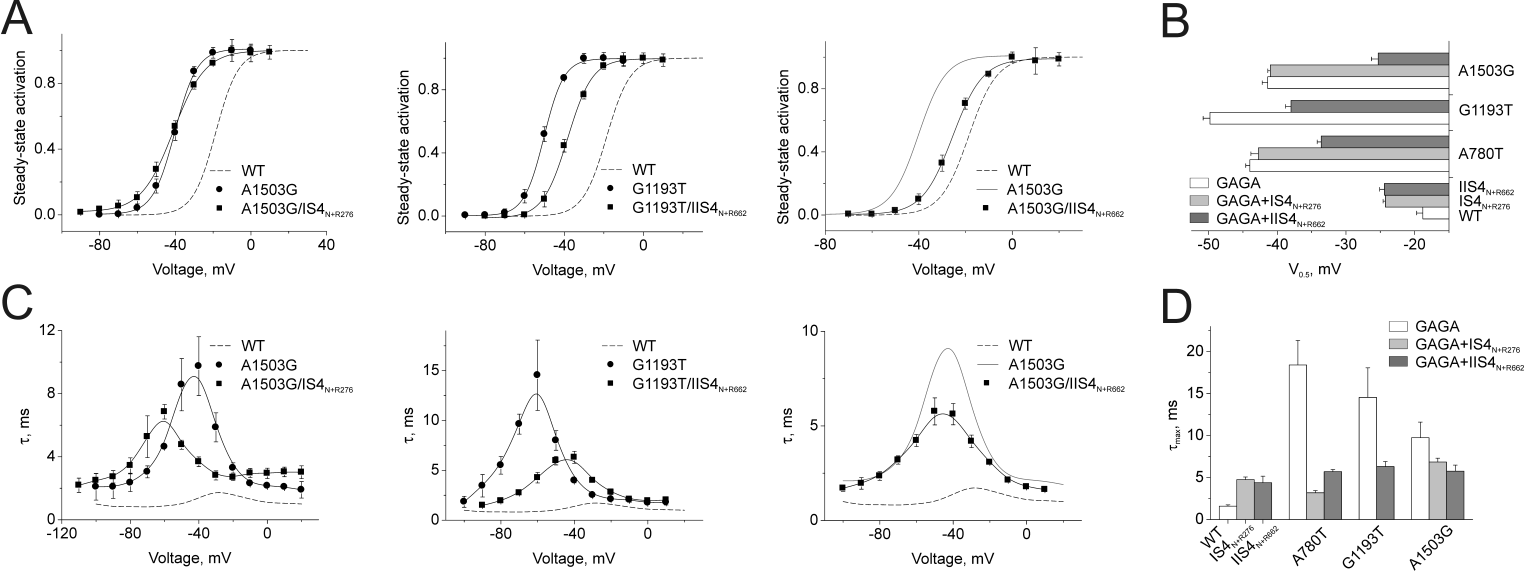


**Fig. S2 Modulation of gating in GAGA-mutants by single charged IS4 or IIS4**

A, averaged activation curves of WT, GAGA-mutants (A1503G and G1193T) and corresponding combinations with single charged IS4 (IS4_N+R276_) or IIS4 (IIS4_N+R662_). Neutralisation of IS4 caused shift of activation curve (*left*), while IIS4 neutralisation caused the rightward shift of activation (see also Table 1).

B, Half-activation voltage of studied constructs. Significant shift of activation caused by GAGA mutations (white bars) is partially rescued by charge neutralisations in IIS4 (dark bars) but not IS4 neutralisation (light bars, see also Table 1).

C and D, voltage-dependent time constants of channel activation/deactivation (C) and τ_max_ (D) of studied constructs. Voltage-sensor neutralisations in IS4 or IIS4 accelerate the “slowly” gating GAGA mutants.

FIGURE S3


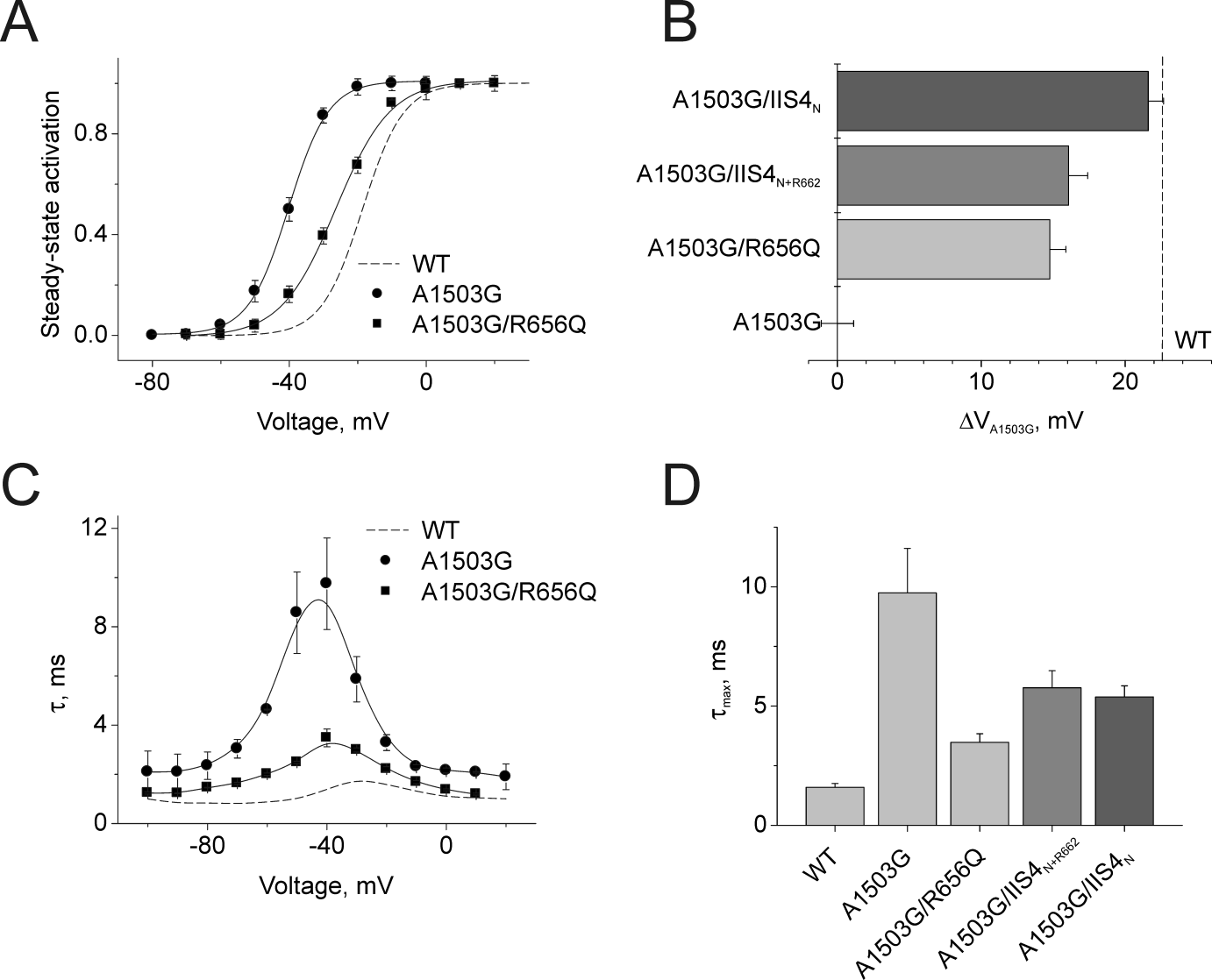


**Figure S3. Charge neutralisation in IIS4 rescues gating disturbances in GAGA position of domain IV.**

A and B, Effect of IIS4 charge neutralisations on steady-state activation of A1503G (segment IVS6). Dashed line represents the position of WT steady-state curve relative to A1503G (see also Table 1).

C and D, voltage-dependent time constants of channel activation/deactivation (C) and τmax (D) of studied constructs.

FIGURE S4


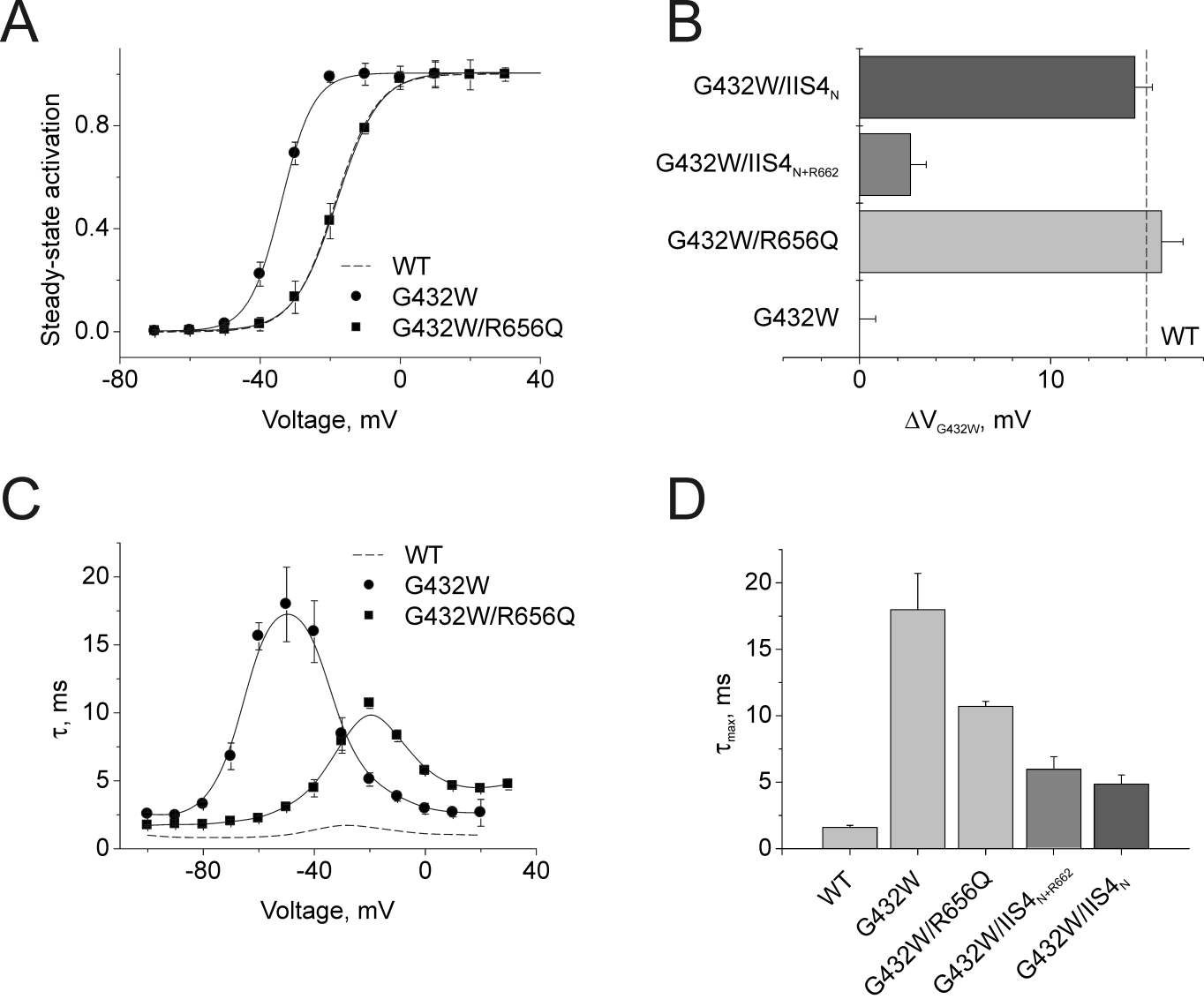


**Fig. S4 Charge neutralisation in domain II (IIS4) rescues gating disturbances in GAGA position of domain I.**

A and B, Effect of IIS4 charge neutralisations on steady-state activation of G432W (segment IS6).. Dashed line represents the position of WT steady-state curve relative to G432W (see also Table 1).

C and D, voltage-dependent time constants of channel activation/deactivation (C) and τmax (D) of studied constructs.
